# *N*-(4-Fluoro­benzo­yl)-*N*′,*N*′′-diisopropyl­phospho­ric triamide

**DOI:** 10.1107/S1600536812046326

**Published:** 2012-11-24

**Authors:** Mehrdad Pourayoubi, Atekeh Tarahhomi, Arnold L. Rheingold, James A. Golen

**Affiliations:** a Department of Chemistry, Ferdowsi University of Mashhad, Mashhad, Iran; b Department of Chemistry, University of California, San Diego, 9500 Gilman Drive, La Jolla, CA 92093, USA

## Abstract

The asymmetric unit of the title phospho­ric triamide, C_13_H_21_FN_3_O_2_P, consists of two independent mol­ecules. In each mol­ecule, the P=O group and the N—H unit belonging to the C(O)NHP(O) fragment are in a *syn* conformation with respect to each other. An intra­molecular N—H⋯O hydrogen bond occurs in each mol­ecule. The P atom adopts a distorted tetra­hedral environment. The methyl groups of an isopropyl fragment are disordered over two sets of sites with refined occupancies of 0.458 (5) and 0.542 (5). In the crystal, mol­ecules are linked through N—H⋯O(=P) and N—H⋯O(=C) hydrogen bonds into chains along [001].

## Related literature

For related structures with a [C(O)NH]P(O)[NHC]_2_ moiety, see: Pourayoubi *et al.* (2011 ▶); Raissi Shabari *et al.* (2012 ▶). For the preparation of the starting compound 4-F—C_6_H_4_C(O)NHP(O)Cl_2_, see: Tarahhomi *et al.* (2011 ▶).
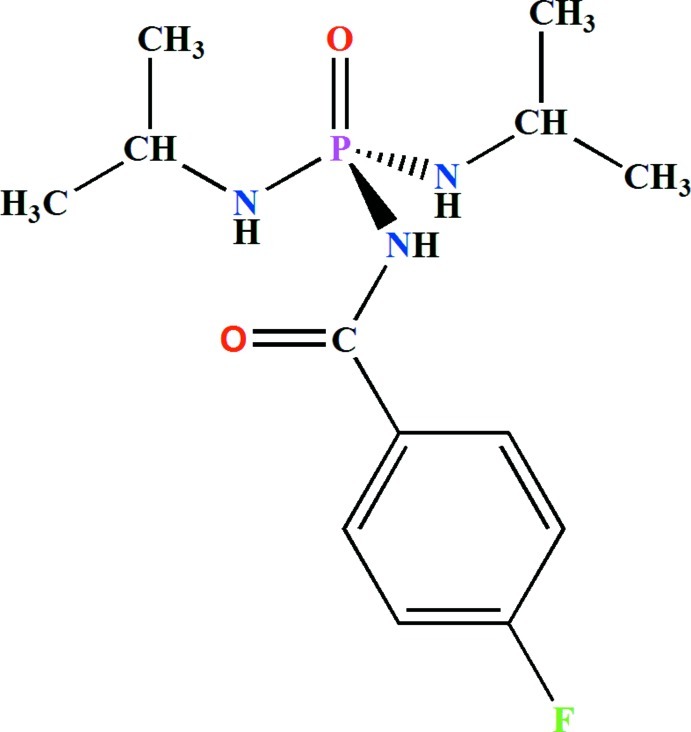


## Experimental

### 

#### Crystal data

C_13_H_21_FN_3_O_2_P
*M* *_r_* = 301.30
Monoclinic, 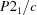

*a* = 15.9974 (12) Å
*b* = 10.7474 (7) Å
*c* = 19.5478 (13) Å
β = 111.461 (2)°
*V* = 3127.8 (4) Å^3^
*Z* = 8
Mo *K*α radiation
μ = 0.19 mm^−1^
*T* = 100 K
0.22 × 0.20 × 0.15 mm

#### Data collection

Bruker APEXII CCD diffractometer
Absorption correction: multi-scan (*SADABS*; Sheldrick, 2004 ▶) *T*_min_ = 0.959, *T*_max_ = 0.972
24038 measured reflections
6366 independent reflections
4979 reflections with *I* > 2σ(*I*)
*R*_int_ = 0.035

#### Refinement

*R*[*F* ^2^ > 2σ(*F* ^2^)] = 0.051
*wR*(*F* ^2^) = 0.140
*S* = 1.08
6366 reflections
385 parameters
11 restraints
H atoms treated by a mixture of independent and constrained refinement
Δρ_max_ = 1.28 e Å^−3^
Δρ_min_ = −1.25 e Å^−3^

### 

Data collection: *APEX2* (Bruker, 2005 ▶); cell refinement: *SAINT* (Bruker, 2005 ▶); data reduction: *SAINT*; program(s) used to solve structure: *SHELXS97* (Sheldrick, 2008 ▶); program(s) used to refine structure: *SHELXL97* (Sheldrick, 2008 ▶); molecular graphics: *Mercury* (Macrae *et al.*, 2008 ▶) and *SHELXTL* (Sheldrick, 2008 ▶); software used to prepare material for publication: *SHELXTL* and *enCIFer* (Allen *et al.*, 2004 ▶).

## Supplementary Material

Crystal structure: contains datablock(s) I, global. DOI: 10.1107/S1600536812046326/ff2089sup1.cif

Structure factors: contains datablock(s) I. DOI: 10.1107/S1600536812046326/ff2089Isup2.hkl

Additional supplementary materials:  crystallographic information; 3D view; checkCIF report

## Figures and Tables

**Table 1:** Hydrogen-bond geometry (Å, °)

*D*—H⋯*A*	*D*—H	H⋯*A*	*D*⋯*A*	*D*—H⋯*A*
N1—H1*N*⋯O4^i^	0.87 (2)	1.97 (2)	2.832 (2)	172 (2)
N2—H2*N*⋯O3^ii^	0.85 (2)	2.18 (2)	3.010 (2)	167 (2)
N3—H3*N*⋯O1	0.83 (2)	2.51 (3)	2.990 (3)	118 (2)
N4—H4*N*⋯O2^iii^	0.85 (2)	1.96 (2)	2.802 (2)	171 (3)
N5—H5*N*⋯O1^ii^	0.85 (2)	2.15 (2)	2.990 (2)	167 (2)
N6—H6*N*⋯O3	0.83 (2)	2.51 (3)	3.055 (3)	124 (2)

Symmetry codes: (i) 
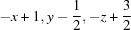
; (ii) 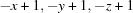; (iii) 
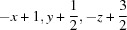
.

**Fig. 1.**
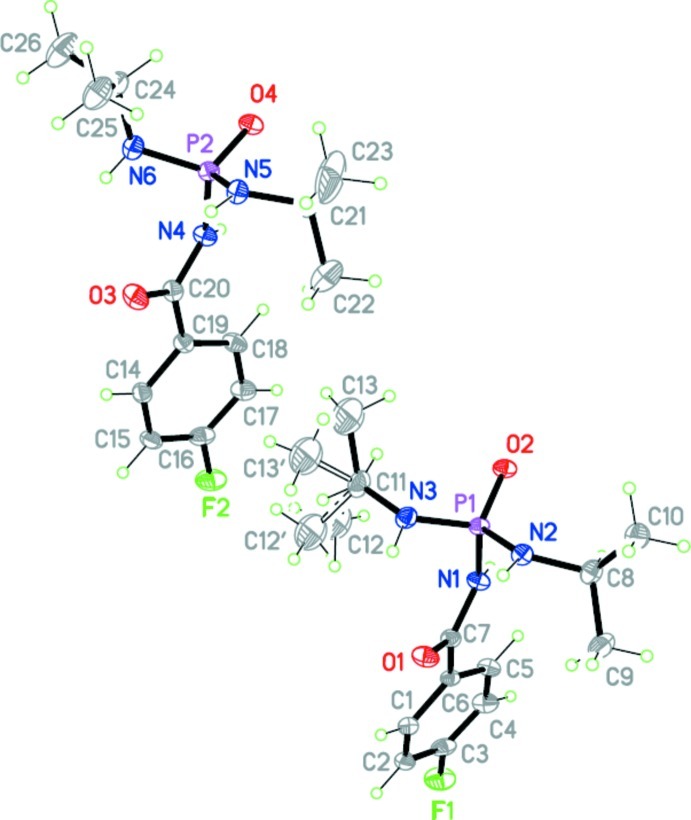
An ORTEP-style plot and atom labeling scheme for the title compound. Displacement ellipsoids are given at 50% probability level and H atoms are drawn as small spheres of arbitrary radii.

**Fig. 2.**
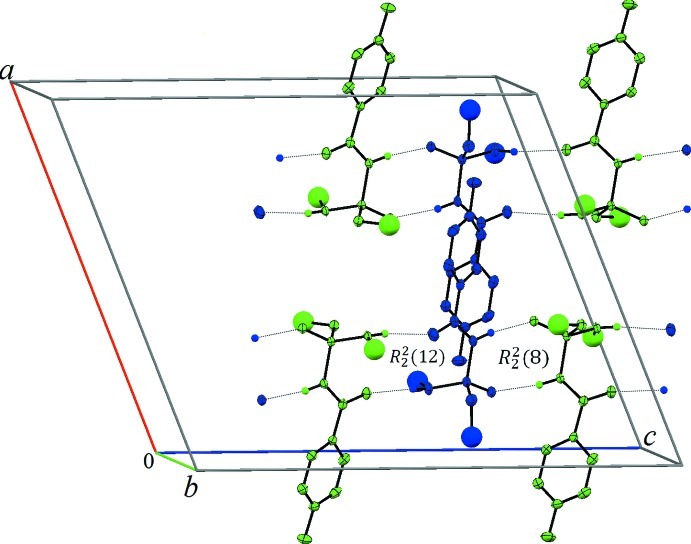
Part of the crystal packing of the title compound via the N—H···O hydrogen bonds parallel to [001], building R22(8) and R22(12) rings. The intermolecular hydrogen bonds are shown as dotted lines and the H atoms not involved in hydrogen bonding have been omitted for the sake of clarity. The CH(CH3)2 substituents are shown as balls. The intramolecular N—H···O hydrogen bonds are not shown.

## supplementary crystallographic information

### Comment

The structure determination of the title compound, [4-F—C_6_H_4_C(O)NH]P(O)[NHCH(CH_3_)_2_]_2_ (Fig. 1), was performed as a part of a project on the synthesis of new phosphoric triamides with a [C(O)NH]P(O)[NHC]_2_ skeleton (Pourayoubi *et al.*, 2011; Raissi Shabari *et al.*, 2012).

The asymmetric unit of the title compound consists of two independent molecules. In one molecule, disorder with respect to the methyl carbon atoms C12 and C13 of an isopropyl group was treated using a two-part model (45.8/54.2) with restrained C—C distances of 1.55 (0.02) Å.

In the C(O)NHP(O) fragment, the phosphoryl group adopts a *syn* orientation with respect to the N—H unit. The P atoms are in a distorted tetrahedral environment as has been noted for other phosphoric triamides (Pourayoubi *et al.*, 2011). The P═O, C═O and P—N bond lengths and the P—N—C bond angles are within the expected values (Pourayoubi *et al.*, 2011; Raissi Shabari *et al.*, 2012).

In the crystal structure, the molecules are linked through N—H···O(═P) and N—H···O(═C) hydrogen bonds into chains along [001], giving *R*_2_^2^(8) and *R*_2_^2^(12) rings (Fig. 2). This sequence of ring motifs is similar to most of the phosphoric triamides with a [C(O)NH]P(O)[NHC]_2_ skeleton (Pourayoubi *et al.*, 2011). Moreover, two intramolecular N—H···O(═C) hydrogen bonds (Table 1) are found in the structure.

### Experimental

4-F—C_6_H_4_C(O)NHP(O)Cl_2_ was prepared according to the literature method reported by Tarahhomi *et al.* (2011).

To a solution of 4-F—C_6_H_4_C(O)NHP(O)Cl_2_ (1 mmol) in chloroform (25 ml), a solution of isopropylamine (4 mmol) in chloroform (5 ml) was added at 273 K. After 4 h stirring, the solvent was removed and the product was washed with distilled water and recrystallized from a mixture of CH_3_OH/DMF (5:1 *v/v*) at room temperature.

### Refinement

All non-hydrogen atoms refined anisotropically by full matrix least squares on F^2^. Disorder with respect to the methyl carbon atoms C12 and C13 of an isopropyl group was treated using a two part model (45.8/54.2) with restrained C—C distances of 1.55 (0.02) Å. Hydrogen atoms H1N, H2N, H3N, H4N, H5N, and H6N were found from a Fourier difference map and were refined isotropically with N—H distances of 0.87 (2) Å and 1.20 *U*_eq_ of parent N atom. All other hydrogen atoms were placed in calculated positions with C—H distances of (C—HAr) 0.95 Å, (C—H) 1.00 Å, (CH_3_) 0.98 Å and *U*_eq_ of 1.20 and 1.50 of parent C atom.

### Crystal data

**Table:**

C_13_H_21_FN_3_O_2_P	*F*(000) = 1280
*M**_r_* = 301.30	*D*_x_ = 1.280 Mg m^−^^3^
Monoclinic, *P*2_1_/*c*	Mo *K*α radiation, λ = 0.71073 Å
*a* = 15.9974 (12) Å	Cell parameters from 6765 reflections
*b* = 10.7474 (7) Å	θ = 2.7–26.4°
*c* = 19.5478 (13) Å	µ = 0.19 mm^−^^1^
β = 111.461 (2)°	*T* = 100 K
*V* = 3127.8 (4) Å^3^	Block, colourless
*Z* = 8	0.22 × 0.20 × 0.15 mm

### Data collection

**Table:**

Bruker APEXII CCD diffractometer	6366 independent reflections
Radiation source: fine-focus sealed tube	4979 reflections with *I* > 2σ(*I*)
Graphite monochromator	*R*_int_ = 0.035
φ and ω scans	θ_max_ = 26.5°, θ_min_ = 2.2°
Absorption correction: multi-scan (*SADABS*; Sheldrick, 2004)	*h* = −20→19
*T*_min_ = 0.959, *T*_max_ = 0.972	*k* = −12→13
24038 measured reflections	*l* = −24→24

### Refinement

**Table:**

Refinement on *F*^2^	Primary atom site location: structure-invariant direct methods
Least-squares matrix: full	Secondary atom site location: difference Fourier map
*R*[*F*^2^ > 2σ(*F*^2^)] = 0.051	Hydrogen site location: inferred from neighbouring sites
*wR*(*F*^2^) = 0.140	H atoms treated by a mixture of independent and constrained refinement
*S* = 1.08	*w* = 1/[σ^2^(*F*_o_^2^) + (0.0604*P*)^2^ + 3.5857*P*] where *P* = (*F*_o_^2^ + 2*F*_c_^2^)/3
6366 reflections	(Δ/σ)_max_ = 0.024
385 parameters	Δρ_max_ = 1.28 e Å^−^^3^
11 restraints	Δρ_min_ = −1.25 e Å^−^^3^

### Special details

**Table:**

Experimental. IR (KBr, ν, cm^-1^): 3345, 3095, 2972, 1657, 1452, 1291, 1215, 1139, 1025, 887, 769, 683.
Geometry. All e.s.d.'s (except the e.s.d. in the dihedral angle between two l.s. planes) are estimated using the full covariance matrix. The cell e.s.d.'s are taken into account individually in the estimation of e.s.d.'s in distances, angles and torsion angles; correlations between e.s.d.'s in cell parameters are only used when they are defined by crystal symmetry. An approximate (isotropic) treatment of cell e.s.d.'s is used for estimating e.s.d.'s involving l.s. planes.
Refinement. Refinement of *F*^2^ against ALL reflections. The weighted *R*-factor *wR* and goodness of fit *S* are based on *F*^2^, conventional *R*-factors *R* are based on *F*, with *F* set to zero for negative *F*^2^. The threshold expression of *F*^2^ > σ(*F*^2^) is used only for calculating *R*-factors(gt) *etc*. and is not relevant to the choice of reflections for refinement. *R*-factors based on *F*^2^ are statistically about twice as large as those based on *F*, and *R*- factors based on ALL data will be even larger.

### Fractional atomic coordinates and isotropic or equivalent isotropic displacement parameters (Å^2^)

**Table:**

	*x*	*y*	*z*	*U*_iso_*/*U*_eq_	Occ. (<1)
P1	0.66641 (4)	0.16703 (5)	0.60938 (3)	0.01446 (15)	
P2	0.21362 (4)	0.66868 (5)	0.64779 (3)	0.01601 (15)	
F1	1.19973 (10)	0.26579 (16)	0.81566 (8)	0.0353 (4)	
F2	0.75090 (10)	0.72695 (16)	0.81958 (8)	0.0346 (4)	
O1	0.81182 (11)	0.27790 (16)	0.57067 (9)	0.0233 (4)	
O2	0.62755 (10)	0.11428 (15)	0.66103 (8)	0.0184 (3)	
O3	0.34880 (12)	0.81368 (16)	0.60767 (9)	0.0257 (4)	
O4	0.18241 (11)	0.59057 (15)	0.69603 (8)	0.0191 (3)	
N1	0.77938 (13)	0.17148 (18)	0.65776 (10)	0.0167 (4)	
H1N	0.7967 (17)	0.148 (2)	0.7034 (10)	0.020*	
N2	0.64876 (13)	0.08672 (17)	0.53551 (10)	0.0163 (4)	
H2N	0.6444 (17)	0.124 (2)	0.4962 (11)	0.020*	
N3	0.63066 (14)	0.30688 (18)	0.58047 (11)	0.0199 (4)	
H3N	0.6526 (18)	0.333 (2)	0.5509 (13)	0.024*	
N4	0.32492 (13)	0.69042 (18)	0.69250 (10)	0.0182 (4)	
H4N	0.3451 (17)	0.668 (2)	0.7372 (10)	0.022*	
N5	0.19346 (14)	0.60247 (18)	0.56892 (10)	0.0190 (4)	
H5N	0.1955 (18)	0.647 (2)	0.5336 (12)	0.023*	
N6	0.17165 (14)	0.80866 (19)	0.63345 (11)	0.0225 (4)	
H6N	0.2011 (18)	0.861 (2)	0.6202 (15)	0.027*	
C1	0.98985 (16)	0.3212 (2)	0.66618 (13)	0.0214 (5)	
H1B	0.9655	0.3738	0.6245	0.026*	
C2	1.07965 (17)	0.3330 (2)	0.71090 (14)	0.0239 (5)	
H2C	1.1172	0.3931	0.7009	0.029*	
C3	1.11214 (16)	0.2545 (2)	0.77028 (13)	0.0240 (5)	
C4	1.06095 (17)	0.1645 (2)	0.78636 (13)	0.0248 (5)	
H4C	1.0865	0.1105	0.8272	0.030*	
C5	0.97113 (16)	0.1547 (2)	0.74148 (12)	0.0204 (5)	
H5B	0.9343	0.0940	0.7519	0.024*	
C6	0.93470 (15)	0.2335 (2)	0.68126 (12)	0.0163 (4)	
C7	0.83791 (15)	0.2292 (2)	0.63192 (12)	0.0174 (5)	
C8	0.67839 (16)	−0.0441 (2)	0.54144 (13)	0.0216 (5)	
H8A	0.6991	−0.0683	0.5944	0.026*	
C9	0.75718 (18)	−0.0592 (3)	0.51657 (15)	0.0314 (6)	
H9A	0.8062	−0.0041	0.5455	0.047*	
H9B	0.7384	−0.0377	0.4644	0.047*	
H9C	0.7779	−0.1458	0.5238	0.047*	
C10	0.59970 (19)	−0.1273 (3)	0.49919 (16)	0.0339 (6)	
H10A	0.5511	−0.1153	0.5178	0.051*	
H10B	0.6190	−0.2145	0.5057	0.051*	
H10C	0.5782	−0.1059	0.4469	0.051*	
C11	0.62386 (19)	0.4006 (2)	0.63394 (14)	0.0293 (6)	
H11A	0.6168	0.4687	0.5974	0.044*	0.458 (5)
H11B	0.6167	0.3669	0.6792	0.044*	0.542 (5)
C12	0.7119 (6)	0.4539 (9)	0.6829 (5)	0.0619 (11)	0.458 (5)
H12A	0.7376	0.4014	0.7266	0.093*	0.458 (5)
H12B	0.7026	0.5382	0.6978	0.093*	0.458 (5)
H12C	0.7532	0.4570	0.6562	0.093*	0.458 (5)
C13	0.5276 (6)	0.4168 (8)	0.6273 (5)	0.0619 (11)	0.458 (5)
H13A	0.4995	0.3349	0.6244	0.093*	0.458 (5)
H13B	0.4948	0.4644	0.5828	0.093*	0.458 (5)
H13C	0.5258	0.4615	0.6704	0.093*	0.458 (5)
C12'	0.6977 (5)	0.5009 (7)	0.6466 (5)	0.0619 (11)	0.542 (5)
H12D	0.7565	0.4650	0.6752	0.093*	0.542 (5)
H12E	0.6857	0.5712	0.6736	0.093*	0.542 (5)
H12F	0.6977	0.5299	0.5991	0.093*	0.542 (5)
C13'	0.5514 (5)	0.4973 (6)	0.5888 (4)	0.0619 (11)	0.542 (5)
H13D	0.4917	0.4590	0.5731	0.093*	0.542 (5)
H13E	0.5639	0.5235	0.5455	0.093*	0.542 (5)
H13F	0.5532	0.5700	0.6196	0.093*	0.542 (5)
C14	0.53551 (17)	0.8319 (2)	0.69340 (13)	0.0209 (5)	
H14A	0.5109	0.8941	0.6570	0.025*	
C15	0.62753 (17)	0.8276 (2)	0.73154 (14)	0.0245 (5)	
H15A	0.6664	0.8872	0.7229	0.029*	
C16	0.66076 (16)	0.7340 (2)	0.78238 (13)	0.0241 (5)	
C17	0.60747 (17)	0.6462 (2)	0.79746 (13)	0.0248 (5)	
H17A	0.6330	0.5823	0.8325	0.030*	
C18	0.51529 (16)	0.6533 (2)	0.75999 (13)	0.0227 (5)	
H18A	0.4770	0.5946	0.7701	0.027*	
C19	0.47844 (16)	0.7459 (2)	0.70771 (12)	0.0179 (5)	
C20	0.37995 (16)	0.7536 (2)	0.66527 (12)	0.0186 (5)	
C21	0.20729 (17)	0.4683 (2)	0.56179 (13)	0.0232 (5)	
H21A	0.2013	0.4257	0.6053	0.028*	
C22	0.2994 (2)	0.4408 (3)	0.5623 (2)	0.0550 (9)	
H22A	0.3444	0.4811	0.6049	0.082*	
H22B	0.3046	0.4728	0.5171	0.082*	
H22C	0.3093	0.3507	0.5652	0.082*	
C23	0.1340 (3)	0.4201 (3)	0.4942 (2)	0.0723 (14)	
H23A	0.0754	0.4378	0.4974	0.108*	
H23B	0.1409	0.3300	0.4904	0.108*	
H23C	0.1378	0.4610	0.4506	0.108*	
C24	0.07352 (18)	0.8274 (2)	0.60296 (14)	0.0277 (6)	
H24A	0.0453	0.7512	0.6145	0.033*	
C25	0.0378 (2)	0.8417 (3)	0.52048 (15)	0.0354 (7)	
H25A	0.0521	0.7670	0.4982	0.053*	
H25B	0.0655	0.9145	0.5072	0.053*	
H25C	−0.0275	0.8529	0.5025	0.053*	
C26	0.0489 (2)	0.9359 (3)	0.64109 (16)	0.0430 (8)	
H26A	0.0694	0.9193	0.6940	0.064*	
H26B	−0.0165	0.9470	0.6214	0.064*	
H26C	0.0777	1.0117	0.6326	0.064*	

### Atomic displacement parameters (Å^2^)

**Table:**

	*U*^11^	*U*^22^	*U*^33^	*U*^12^	*U*^13^	*U*^23^
P1	0.0147 (3)	0.0187 (3)	0.0103 (3)	−0.0003 (2)	0.0049 (2)	−0.0013 (2)
P2	0.0177 (3)	0.0191 (3)	0.0117 (3)	0.0005 (2)	0.0059 (2)	0.0002 (2)
F1	0.0164 (8)	0.0524 (10)	0.0328 (8)	−0.0072 (7)	0.0038 (6)	−0.0086 (7)
F2	0.0178 (8)	0.0483 (10)	0.0357 (9)	−0.0050 (7)	0.0076 (6)	−0.0064 (7)
O1	0.0236 (9)	0.0325 (9)	0.0150 (8)	−0.0006 (7)	0.0085 (7)	0.0053 (7)
O2	0.0179 (8)	0.0257 (8)	0.0128 (7)	−0.0031 (7)	0.0070 (6)	−0.0031 (6)
O3	0.0286 (10)	0.0303 (9)	0.0171 (8)	−0.0017 (7)	0.0072 (7)	0.0081 (7)
O4	0.0197 (9)	0.0259 (9)	0.0128 (7)	−0.0032 (7)	0.0070 (6)	−0.0004 (6)
N1	0.0157 (10)	0.0243 (10)	0.0096 (8)	−0.0030 (8)	0.0039 (7)	0.0006 (7)
N2	0.0196 (10)	0.0201 (10)	0.0086 (8)	0.0011 (8)	0.0044 (7)	0.0010 (7)
N3	0.0234 (11)	0.0206 (10)	0.0172 (10)	0.0028 (8)	0.0093 (8)	0.0005 (8)
N4	0.0191 (10)	0.0253 (10)	0.0097 (9)	−0.0015 (8)	0.0047 (7)	0.0034 (7)
N5	0.0265 (11)	0.0193 (10)	0.0123 (9)	0.0025 (8)	0.0084 (8)	0.0021 (7)
N6	0.0251 (12)	0.0198 (10)	0.0228 (10)	0.0026 (8)	0.0091 (9)	−0.0005 (8)
C1	0.0225 (13)	0.0202 (12)	0.0264 (12)	0.0010 (9)	0.0146 (10)	0.0008 (9)
C2	0.0213 (13)	0.0214 (12)	0.0341 (14)	−0.0048 (10)	0.0163 (11)	−0.0051 (10)
C3	0.0148 (12)	0.0341 (14)	0.0235 (12)	−0.0031 (10)	0.0076 (9)	−0.0100 (10)
C4	0.0206 (13)	0.0354 (14)	0.0178 (11)	0.0021 (11)	0.0063 (10)	0.0037 (10)
C5	0.0179 (12)	0.0259 (12)	0.0187 (11)	−0.0023 (9)	0.0082 (9)	0.0013 (9)
C6	0.0159 (11)	0.0201 (11)	0.0147 (10)	−0.0008 (9)	0.0079 (9)	−0.0025 (8)
C7	0.0201 (12)	0.0192 (11)	0.0152 (11)	−0.0008 (9)	0.0089 (9)	−0.0007 (9)
C8	0.0252 (13)	0.0219 (12)	0.0169 (11)	0.0032 (10)	0.0067 (9)	−0.0015 (9)
C9	0.0239 (14)	0.0318 (14)	0.0384 (15)	0.0035 (11)	0.0112 (12)	−0.0096 (12)
C10	0.0354 (16)	0.0271 (14)	0.0448 (17)	−0.0065 (12)	0.0215 (13)	−0.0123 (12)
C11	0.0413 (16)	0.0229 (13)	0.0277 (13)	0.0037 (11)	0.0173 (12)	−0.0055 (10)
C12	0.083 (3)	0.046 (2)	0.070 (3)	−0.003 (2)	0.044 (2)	−0.0214 (19)
C13	0.083 (3)	0.046 (2)	0.070 (3)	−0.003 (2)	0.044 (2)	−0.0214 (19)
C12'	0.083 (3)	0.046 (2)	0.070 (3)	−0.003 (2)	0.044 (2)	−0.0214 (19)
C13'	0.083 (3)	0.046 (2)	0.070 (3)	−0.003 (2)	0.044 (2)	−0.0214 (19)
C14	0.0285 (13)	0.0173 (11)	0.0218 (12)	−0.0018 (10)	0.0151 (10)	−0.0013 (9)
C15	0.0243 (13)	0.0227 (12)	0.0329 (13)	−0.0075 (10)	0.0179 (11)	−0.0054 (10)
C16	0.0173 (12)	0.0326 (13)	0.0232 (12)	−0.0034 (10)	0.0082 (10)	−0.0093 (10)
C17	0.0234 (13)	0.0334 (14)	0.0185 (11)	0.0007 (10)	0.0088 (10)	0.0034 (10)
C18	0.0214 (13)	0.0295 (13)	0.0183 (11)	−0.0033 (10)	0.0085 (10)	0.0041 (10)
C19	0.0225 (12)	0.0200 (11)	0.0139 (10)	−0.0019 (9)	0.0099 (9)	−0.0023 (8)
C20	0.0242 (13)	0.0186 (11)	0.0140 (10)	−0.0005 (9)	0.0081 (9)	−0.0002 (9)
C21	0.0321 (14)	0.0202 (12)	0.0188 (11)	0.0048 (10)	0.0111 (10)	−0.0010 (9)
C22	0.053 (2)	0.0428 (19)	0.080 (3)	0.0130 (16)	0.037 (2)	−0.0067 (18)
C23	0.061 (2)	0.0353 (18)	0.087 (3)	0.0087 (17)	−0.013 (2)	−0.0311 (19)
C24	0.0289 (14)	0.0278 (13)	0.0281 (13)	0.0090 (11)	0.0124 (11)	0.0041 (10)
C25	0.0352 (16)	0.0377 (15)	0.0277 (14)	0.0128 (12)	0.0048 (12)	0.0006 (12)
C26	0.053 (2)	0.0476 (18)	0.0314 (15)	0.0243 (15)	0.0183 (14)	0.0018 (13)

### Geometric parameters (Å, º)

**Table:**

P1—O2	1.4788 (16)	C11—C12'	1.551 (7)
P1—N2	1.6159 (18)	C11—C13'	1.567 (7)
P1—N3	1.634 (2)	C11—H11A	1.0000
P1—N1	1.7065 (19)	C11—H11B	1.0000
P2—O4	1.4792 (16)	C12—H12A	0.9800
P2—N5	1.6197 (19)	C12—H12B	0.9800
P2—N6	1.629 (2)	C12—H12C	0.9800
P2—N4	1.688 (2)	C13—H13A	0.9800
F1—C3	1.362 (3)	C13—H13B	0.9800
F2—C16	1.359 (3)	C13—H13C	0.9800
O1—C7	1.231 (3)	C12'—H12D	0.9800
O3—C20	1.234 (3)	C12'—H12E	0.9800
N1—C7	1.365 (3)	C12'—H12F	0.9800
N1—H1N	0.868 (16)	C13'—H13D	0.9800
N2—C8	1.475 (3)	C13'—H13E	0.9800
N2—H2N	0.848 (16)	C13'—H13F	0.9800
N3—C11	1.484 (3)	C14—C15	1.386 (4)
N3—H3N	0.828 (17)	C14—C19	1.397 (3)
N4—C20	1.364 (3)	C14—H14A	0.9500
N4—H4N	0.849 (17)	C15—C16	1.377 (4)
N5—C21	1.473 (3)	C15—H15A	0.9500
N5—H5N	0.852 (17)	C16—C17	1.374 (4)
N6—C24	1.475 (3)	C17—C18	1.388 (3)
N6—H6N	0.833 (17)	C17—H17A	0.9500
C1—C2	1.388 (3)	C18—C19	1.393 (3)
C1—C6	1.395 (3)	C18—H18A	0.9500
C1—H1B	0.9500	C19—C20	1.490 (3)
C2—C3	1.374 (4)	C21—C22	1.498 (4)
C2—H2C	0.9500	C21—C23	1.502 (4)
C3—C4	1.375 (4)	C21—H21A	1.0000
C4—C5	1.387 (3)	C22—H22A	0.9800
C4—H4C	0.9500	C22—H22B	0.9800
C5—C6	1.393 (3)	C22—H22C	0.9800
C5—H5B	0.9500	C23—H23A	0.9800
C6—C7	1.496 (3)	C23—H23B	0.9800
C8—C9	1.516 (4)	C23—H23C	0.9800
C8—C10	1.519 (4)	C24—C25	1.509 (4)
C8—H8A	1.0000	C24—C26	1.512 (4)
C9—H9A	0.9800	C24—H24A	1.0000
C9—H9B	0.9800	C25—H25A	0.9800
C9—H9C	0.9800	C25—H25B	0.9800
C10—H10A	0.9800	C25—H25C	0.9800
C10—H10B	0.9800	C26—H26A	0.9800
C10—H10C	0.9800	C26—H26B	0.9800
C11—C12	1.497 (9)	C26—H26C	0.9800
C11—C13	1.508 (8)		
			
O2—P1—N2	115.19 (10)	C13—C11—H11B	71.0
O2—P1—N3	114.04 (10)	C12'—C11—H11B	116.0
N2—P1—N3	104.94 (10)	C13'—C11—H11B	116.0
O2—P1—N1	104.88 (9)	H11A—C11—H11B	151.4
N2—P1—N1	108.28 (10)	C11—C12—H12A	109.5
N3—P1—N1	109.37 (10)	C11—C12—H12B	109.5
O4—P2—N5	111.19 (10)	H12A—C12—H12B	109.5
O4—P2—N6	114.82 (10)	C11—C12—H12C	109.5
N5—P2—N6	108.42 (10)	H12A—C12—H12C	109.5
O4—P2—N4	106.65 (9)	H12B—C12—H12C	109.5
N5—P2—N4	111.01 (10)	C11—C13—H13A	109.5
N6—P2—N4	104.57 (11)	C11—C13—H13B	109.5
C7—N1—P1	122.57 (16)	H13A—C13—H13B	109.5
C7—N1—H1N	120.4 (17)	C11—C13—H13C	109.5
P1—N1—H1N	115.9 (17)	H13A—C13—H13C	109.5
C8—N2—P1	119.54 (15)	H13B—C13—H13C	109.5
C8—N2—H2N	116.2 (18)	C11—C12'—H12D	109.5
P1—N2—H2N	119.1 (18)	C11—C12'—H12E	109.5
C11—N3—P1	119.18 (16)	H12D—C12'—H12E	109.5
C11—N3—H3N	114.6 (19)	C11—C12'—H12F	109.5
P1—N3—H3N	112.0 (19)	H12D—C12'—H12F	109.5
C20—N4—P2	125.15 (16)	H12E—C12'—H12F	109.5
C20—N4—H4N	119.2 (18)	C11—C13'—H13D	109.5
P2—N4—H4N	115.2 (18)	C11—C13'—H13E	109.5
C21—N5—P2	122.39 (16)	H13D—C13'—H13E	109.5
C21—N5—H5N	115.1 (18)	C11—C13'—H13F	109.5
P2—N5—H5N	118.0 (18)	H13D—C13'—H13F	109.5
C24—N6—P2	120.41 (17)	H13E—C13'—H13F	109.5
C24—N6—H6N	115 (2)	C15—C14—C19	120.9 (2)
P2—N6—H6N	116 (2)	C15—C14—H14A	119.6
C2—C1—C6	121.2 (2)	C19—C14—H14A	119.6
C2—C1—H1B	119.4	C16—C15—C14	117.8 (2)
C6—C1—H1B	119.4	C16—C15—H15A	121.1
C3—C2—C1	117.4 (2)	C14—C15—H15A	121.1
C3—C2—H2C	121.3	F2—C16—C17	117.8 (2)
C1—C2—H2C	121.3	F2—C16—C15	118.7 (2)
F1—C3—C2	118.4 (2)	C17—C16—C15	123.4 (2)
F1—C3—C4	118.1 (2)	C16—C17—C18	118.1 (2)
C2—C3—C4	123.5 (2)	C16—C17—H17A	120.9
C3—C4—C5	118.3 (2)	C18—C17—H17A	120.9
C3—C4—H4C	120.8	C17—C18—C19	120.6 (2)
C5—C4—H4C	120.8	C17—C18—H18A	119.7
C4—C5—C6	120.3 (2)	C19—C18—H18A	119.7
C4—C5—H5B	119.8	C18—C19—C14	119.1 (2)
C6—C5—H5B	119.8	C18—C19—C20	121.7 (2)
C5—C6—C1	119.2 (2)	C14—C19—C20	119.2 (2)
C5—C6—C7	122.7 (2)	O3—C20—N4	120.9 (2)
C1—C6—C7	118.0 (2)	O3—C20—C19	121.9 (2)
O1—C7—N1	121.2 (2)	N4—C20—C19	117.19 (19)
O1—C7—C6	121.1 (2)	N5—C21—C22	111.9 (2)
N1—C7—C6	117.63 (19)	N5—C21—C23	109.0 (2)
N2—C8—C9	111.0 (2)	C22—C21—C23	112.8 (3)
N2—C8—C10	109.9 (2)	N5—C21—H21A	107.6
C9—C8—C10	112.5 (2)	C22—C21—H21A	107.6
N2—C8—H8A	107.7	C23—C21—H21A	107.6
C9—C8—H8A	107.7	C21—C22—H22A	109.5
C10—C8—H8A	107.7	C21—C22—H22B	109.5
C8—C9—H9A	109.5	H22A—C22—H22B	109.5
C8—C9—H9B	109.5	C21—C22—H22C	109.5
H9A—C9—H9B	109.5	H22A—C22—H22C	109.5
C8—C9—H9C	109.5	H22B—C22—H22C	109.5
H9A—C9—H9C	109.5	C21—C23—H23A	109.5
H9B—C9—H9C	109.5	C21—C23—H23B	109.5
C8—C10—H10A	109.5	H23A—C23—H23B	109.5
C8—C10—H10B	109.5	C21—C23—H23C	109.5
H10A—C10—H10B	109.5	H23A—C23—H23C	109.5
C8—C10—H10C	109.5	H23B—C23—H23C	109.5
H10A—C10—H10C	109.5	N6—C24—C25	112.2 (2)
H10B—C10—H10C	109.5	N6—C24—C26	109.7 (2)
N3—C11—C12	114.5 (4)	C25—C24—C26	112.5 (2)
N3—C11—C13	110.7 (4)	N6—C24—H24A	107.4
C12—C11—C13	134.7 (5)	C25—C24—H24A	107.4
N3—C11—C12'	109.7 (3)	C26—C24—H24A	107.4
C12—C11—C12'	31.6 (4)	C24—C25—H25A	109.5
C13—C11—C12'	129.0 (4)	C24—C25—H25B	109.5
N3—C11—C13'	106.5 (3)	H25A—C25—H25B	109.5
C12—C11—C13'	115.8 (5)	C24—C25—H25C	109.5
C13—C11—C13'	49.7 (4)	H25A—C25—H25C	109.5
C12'—C11—C13'	89.6 (4)	H25B—C25—H25C	109.5
N3—C11—H11A	90.6	C24—C26—H26A	109.5
C12—C11—H11A	90.6	C24—C26—H26B	109.5
C13—C11—H11A	90.6	H26A—C26—H26B	109.5
C12'—C11—H11A	59.1	C24—C26—H26C	109.5
C13'—C11—H11A	41.1	H26A—C26—H26C	109.5
N3—C11—H11B	116.0	H26B—C26—H26C	109.5
C12—C11—H11B	87.7		
			
O2—P1—N1—C7	−171.46 (18)	C5—C6—C7—O1	−163.0 (2)
N2—P1—N1—C7	65.1 (2)	C1—C6—C7—O1	17.6 (3)
N3—P1—N1—C7	−48.8 (2)	C5—C6—C7—N1	18.1 (3)
O2—P1—N2—C8	−57.3 (2)	C1—C6—C7—N1	−161.2 (2)
N3—P1—N2—C8	176.46 (17)	P1—N2—C8—C9	−109.6 (2)
N1—P1—N2—C8	59.74 (19)	P1—N2—C8—C10	125.23 (19)
O2—P1—N3—C11	43.7 (2)	P1—N3—C11—C12	75.4 (5)
N2—P1—N3—C11	170.64 (18)	P1—N3—C11—C13	−102.8 (4)
N1—P1—N3—C11	−73.4 (2)	P1—N3—C11—C12'	109.2 (4)
O4—P2—N4—C20	177.25 (18)	P1—N3—C11—C13'	−155.2 (3)
N5—P2—N4—C20	56.0 (2)	C19—C14—C15—C16	1.7 (3)
N6—P2—N4—C20	−60.7 (2)	C14—C15—C16—F2	179.0 (2)
O4—P2—N5—C21	−41.3 (2)	C14—C15—C16—C17	−0.6 (4)
N6—P2—N5—C21	−168.44 (19)	F2—C16—C17—C18	179.6 (2)
N4—P2—N5—C21	77.2 (2)	C15—C16—C17—C18	−0.8 (4)
O4—P2—N6—C24	−56.2 (2)	C16—C17—C18—C19	1.1 (4)
N5—P2—N6—C24	68.8 (2)	C17—C18—C19—C14	0.0 (3)
N4—P2—N6—C24	−172.75 (17)	C17—C18—C19—C20	178.3 (2)
C6—C1—C2—C3	0.4 (3)	C15—C14—C19—C18	−1.4 (3)
C1—C2—C3—F1	−178.9 (2)	C15—C14—C19—C20	−179.8 (2)
C1—C2—C3—C4	1.2 (4)	P2—N4—C20—O3	9.1 (3)
F1—C3—C4—C5	178.3 (2)	P2—N4—C20—C19	−170.58 (16)
C2—C3—C4—C5	−1.8 (4)	C18—C19—C20—O3	−161.9 (2)
C3—C4—C5—C6	0.8 (4)	C14—C19—C20—O3	16.4 (3)
C4—C5—C6—C1	0.8 (3)	C18—C19—C20—N4	17.7 (3)
C4—C5—C6—C7	−178.6 (2)	C14—C19—C20—N4	−164.0 (2)
C2—C1—C6—C5	−1.4 (3)	P2—N5—C21—C22	−92.0 (3)
C2—C1—C6—C7	178.0 (2)	P2—N5—C21—C23	142.6 (3)
P1—N1—C7—O1	−2.4 (3)	P2—N6—C24—C25	−94.2 (2)
P1—N1—C7—C6	176.42 (15)	P2—N6—C24—C26	140.0 (2)

### Hydrogen-bond geometry (Å, º)

**Table:**

*D*—H···*A*	*D*—H	H···*A*	*D*···*A*	*D*—H···*A*
N1—H1*N*···O4^i^	0.87 (2)	1.97 (2)	2.832 (2)	172 (2)
N2—H2*N*···O3^ii^	0.85 (2)	2.18 (2)	3.010 (2)	167 (2)
N3—H3*N*···O1	0.83 (2)	2.51 (3)	2.990 (3)	118 (2)
N4—H4*N*···O2^iii^	0.85 (2)	1.96 (2)	2.802 (2)	171 (3)
N5—H5*N*···O1^ii^	0.85 (2)	2.15 (2)	2.990 (2)	167 (2)
N6—H6*N*···O3	0.83 (2)	2.51 (3)	3.055 (3)	124 (2)

Symmetry codes: (i) −*x*+1, *y*−1/2, −*z*+3/2; (ii) −*x*+1, −*y*+1, −*z*+1; (iii) −*x*+1, *y*+1/2, −*z*+3/2.
